# Ligand-assisted morphology regulation of AuNi bimetallic nanocrystals for efficient hydrogen evolution†

**DOI:** 10.1039/d2ra06325e

**Published:** 2023-01-04

**Authors:** Chu Zhang, Can Xue

**Affiliations:** a School of Materials Science and Engineering, Nanyang Technological University 50 Nanyang Avenue 639798 Singapore chu012@e.ntu.edu.sg

## Abstract

We report the controllable synthesis of AuNi core–shell (c-AuNi) and Janus (j-AuNi) nanocrystals (NCs) with uniform shape, tunable size and compositions in the presence of trioctylphosphine (TOP) or triphenylphosphine (TPP). The morphology of the AuNi bimetallic NCs could be regulated by varying the structure and concentration of phosphine ligands. The ligand-directed structural evolution mechanism of AuNi bimetallic NCs was investigated and discussed in detail. When loaded on graphitic carbon nitride (GCN) for photocatalytic hydrogen generation, the obtained j-AuNi NCs showed much higher activity for hydrogen evolution than the monometallic (Au and Ni) counterparts, owing to the synergistic effect of plasmon enhanced light absorption from the Au portion and additional electron sink effect from the Ni portion. This work provides a promising route for preparing low-cost Au-based bimetallic catalysts with controllable morphologies and high activities for hydrogen production.

## Abstract 2

Bimetallic AuNi Janus and core–shell nanocrystals were synthesized by varying the structure and concentration of phosphine ligands, and showed excellent H_2_ evolution activity when loaded on graphitic carbon nitride for photocatalytic H_2_ generation.

## Graphical abstract

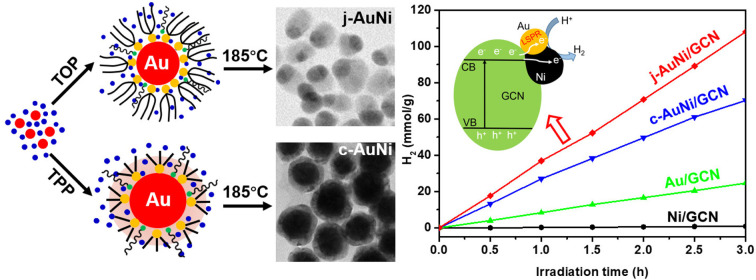

## Introduction

Gold nanoparticles (NPs) have received tremendous interest in the field of catalysis and photocatalysis due to their excellent optical properties and catalytic activities for the hydrogen evolution reaction (HER).^1^ Nevertheless, the high cost and rarity of Au severely limit its industrial application. In order to overcome this problem, incorporating a second earth-abundant metal (*e.g.* Fe, Co, Ni) into Au NCs becomes a promising strategy to reduce the usage of Au.^2–6^ More importantly, in many cases, there is a great enhancement in the catalytic activity or selectivity for bimetal–semiconductor heterostructures owing to the synergistic effect of two metal elements.^7^ For example, Xia's group reported that the Au–Ni/Co supported layered double hydroxides showed not only higher activity but also enhanced selectivity for the hydrogenation of crotonaldehyde to crotonyl alcohol under visible light irradiation.^8^ Ro and co-workers showed that the catalytic activity of AuMo/SiO_2_ was much higher than that of Au/SiO_2_ in the reverse water gas shift reaction under both dark and light conditions.^9^ More recently, Zhang and co-workers demonstrated that Ni@Au nanoparticles (NPs) supported on SiO_2_ exhibited highly selective CO production in CO_2_ hydrogenation because of the formation of NiAu alloy surface at elevated reaction temperature.^10^ As such, due to the unprecedented activities of bimetallic nano-catalysts, tremendous efforts have been devoted to various binary metal–semiconductor heterostructures catalysts over the past decade.^11–17^

Among all the Au-based bimetallic nanostructures, AuNi NCs are of great importance because of their superior performance in different catalytic applications, including CO_2_ reduction, CO oxidation, hydrogen generation, and decomposition of organic wastes.^18–20^ However, due to the large difference in lattice mismatch (13.6%) and redox potential between Au and Ni, there are limited reports on the synthesis of monodisperse AuNi NCs, especially with well-defined architectures.^21–27^ Tsuji and co-workers synthesized conformal Au@Ni core–shell NPs by using Au seed-mediated reduction method in polyol solvents.^28^ Wang *et al.* reported the synthesis of AuNi spindly NPs in octadecylamine through a one-pot noble-metal-induced reduction process.^29^ Duan and co-workers prepared AuNi NPs with different nanostructures by the co-reduction of two metal precursors in oleylamine.^30^ In previous attempts, the precise shape control was known as a challenging issue, while it is a prerequisite to explore the relationship between the structure and catalytic performance. Indeed, many studies have demonstrated that the morphology of bimetallic species could play an important role in the activity, selectivity, and durability of the whole catalysts.^31^

Herein, to explore the effect of geometric architectures of bimetallic AuNi nanocrystals on their catalytic activities for HER under light irradiation, core–shell and Janus AuNi NCs were synthesized *via* a facile one-pot approach in the presence of triphenylphosphine (TPP) and trioctylphosphine (TOP), respectively. The effect of synthetic parameters, such as precursor ratio and ligand concentration, were systematically investigated. Further, the obtained AuNi NCs were loaded as HER catalysts on graphitic carbon nitride (GCN) to explore their activities in photocatalytic hydrogen production under visible light irradiation. The Janus AuNi/GCN nanohybrids exhibited excellent activities for visible-light-driven H_2_ generation due to the positive synergistic effect of Au and Ni metals.

## Experimental

### Chemicals

Gold chloride hydrate (HAuCl_4_·3H_2_O), nickel acetylacetonate (Ni(acac)_2_), urea, oleylamine (OAm), triphenylphosphine (TPP), trioctylphosphine (TOP), triethanolamine (TEOA), sodium sulphate, ethanol, hexane, *N*,*N*-dimethylformamide (DMF) were all purchased from Sigma-Aldrich. All chemicals were used directly without any further purification. Deionized water (18.2 kΩ cm, Milli-Q System, Millipore, Billerica) was used for all experiments. All glasswares were washed with aqua regia and deionized water prior to use.

### Synthesis of core–shell AuNi NCs (c-AuNi)

In a typical synthesis, 0.4 mmol Ni(acac)_2_, 0.4 mmol TPP and 0.1 mmol HAuCl_4_ were dispersed in 0.2 mL ethanol. The mixture was then completely dissolved in 7 mL OAm at 60 °C. After stirring for 10 min, the mixed solution was heated up to 185 °C at a heating rate of ∼5 °C min^−1^ and kept stirring for 30 min at this temperature. After cooling down naturally, the product was precipitated upon addition of ethanol and collected *via* centrifugation. After washed with the mixture of hexane and ethanol, the product powder was redispersed in hexane.

### Synthesis of Janus AuNi NCs (j-AuNi)

In a typical synthesis, 0.4 mmol Ni(acac)_2_ was dissolved in 7 mL of OAm at 120 °C, and then 0.1 mmol of HAuCl_4_ was added into the resulted solution. The mixed solution was kept stirring at 120 °C for 30 min to enable complete reduction of Au^3+^ ions into Au nanoparticle seeds. After that, 0.4 mmol of TOP was added into above solution. Subsequently, the mixed solution was heated up to 185 °C at a heating rate of ∼5 °C min^−1^ and was stirred for 1 h at this temperature. After cooling down naturally, the product was precipitated upon addition of ethanol and collected *via* centrifugation. After washed with the mixture of hexane and ethanol, the product powder was redispersed in hexane.

### Synthesis of g-C_3_N_4_ nanosheets (GCN)

The graphitic carbon nitride was prepared according to previous reports.^32^ Briefly, 20 g urea was heated in air at 550 °C for 4 h at a ramping rate of 5 °C min^−1^. Then, the obtained product was ground into fine powder and heated again at 500 °C for another 4 h with a heating rate of 5 °C min^−1^. Finally, the obtained product was denoted as GCN.

### Loading of AuNi NCs on GCN

Typically, 50 mg GCN was dispersed in 10 ml DMF, and then sonicated for 30 min. Then, 1 ml DMF solution containing 1.5 mg (3 wt%) as-prepared AuNi NCs was added into above suspension. The mixture was sonicated for 30 min and then stirred for 1 h at room temperature. After that, the hybrid powder was collected through gradually removing the solvent *via* a rotary evaporator. After drying, the obtained powder was denoted as AuNi/GCN. Similar procedures were also applied for preparation of Ni/GCN and Au/GCN.

### Characterizations

The powder X-ray diffraction (XRD) patterns of samples were collected by XRD-Bruker D8 Advance Powder (Cu Kα radiation, *λ* = 1.54060 Å), operating at a voltage of 40 kV and a current of 30 mA. The morphologies and structures of samples were studied through transmission electron microscopy (TEM) and scanning transmission electron microscopy (STEM) using the JEOL JEM-2100F at 200 kV. The element distribution and composition of samples were analysed through an energy dispersive X-ray spectrometer (EDS) attached on TEM. The surface composition and electronic state were measured by X-ray photoelectron spectroscopy (XPS, Al Kα source, 1486.6 eV monochromatic X-ray source, Shimadzu Kratos Axis Supra). The binding energies were calibrated with the C 1s binding energy of 284.8 eV. UV-visible diffuse reflectance spectra (DRS) were performed on a Lambda 750 spectrophotometer (PerkinElmer, USA) using BaSO_4_ as reference. Electrochemical measurements were carried out on an electrochemical analyser (Autolab) in 0.2 M aqueous solution of Na_2_SO_4_ (pH 6.8) by using a typical three-electrode cell, including an Ag/AgCl reference electrode (3 M KCl), a Pt plate as the counter electrode and a piece of fluorine-doped tin oxide (FTO) glass coated with samples as the working electrode.

### Photocatalytic hydrogen generation

In a typical process, 10 mg samples of as-synthesized metal NCs-loaded GCN were dispersed in 10 ml aqueous solution of 10 vol% TEOA with sonication. The suspension was then transferred into a glass vessel (20 ml). Then, the vessel was purged with Ar flow for 15 min to remove the residual air. After degassing, the vessel was irradiated with a 300 W Xenon lamp (MAX-302, Asahi Spectra Company, Japan.) coupled with a cut-off filter (*λ* > 420 nm, light intensity of 250 mW cm^−2^). During the reaction, 0.5 ml gas sample was periodically withdrawn from the vessel and analysed by a gas chromatograph (GC, Agilent 7890A) equipped with a thermal conductivity detector (TCD). The apparent quantum yield (AQY) for H_2_ generation was measured under similar experimental conditions by using band-pass filters of 420, 440, 500, and 550 nm, respectively. The AQE was calculated as follow:
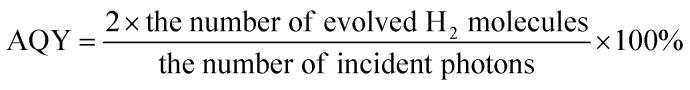


## Results and discussions

### Morphological and structural characterizations

**Fig. 1:**
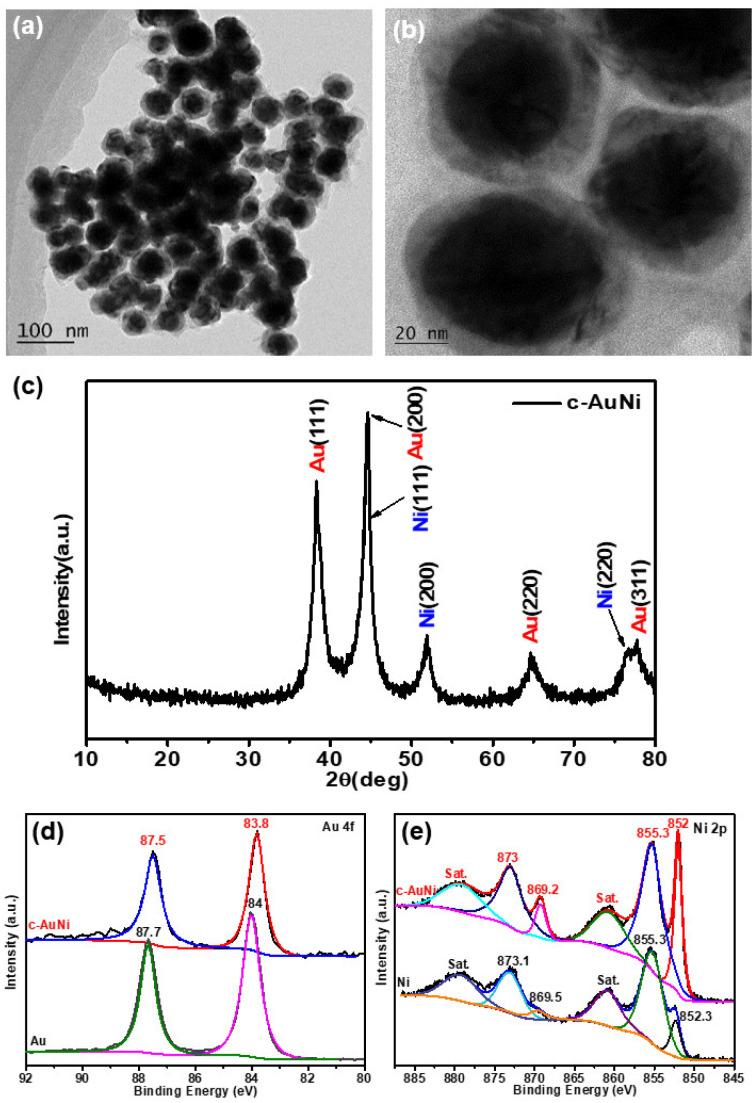
(a and b) TEM image, and (c) XRD pattern of c-AuNi NCs, (d) Au 4f XPS spectra, and (e) Ni 2p XPS spectra of as-synthesized Au NPs, Ni NPs and c-AuNi NCs.

Fig. 1a and b show the typical TEM images of the c-AuNi sample obtained by using TPP as the passivation ligand. The clear contrast between the core and shell of each particle indicates the formation of core–shell nanostructures with an average core size of ∼35 nm and an average shell thickness of ∼10 nm. The EDS mapping image (Fig. S1b†) and line scan information (Fig. S1c†) reveal that the core is Au and the shell is Ni. The molar ratio of Ni to Au was estimated as 4 : 1 based on the quantitative EDS analysis result. The XRD pattern (Fig. 1c) presented faced-centered-cubic (fcc) Au (JCPDS 65-8601) and fcc Ni (JCPDS 65-2865), co-existing in the obtained c-AuNi NCs.

Furthermore, the XPS spectra of c-AuNi NCs were compared with that of individual Au NPs and Ni NPs synthesized under similar conditions (see ESI†). The binding energy values at 852.3 eV and 855.3 eV shown in Fig. 1e were considered as the peaks of metallic nickel and nickel hydroxide, respectively.^33^ It is worth noting that nickel hydroxide was the main phase in pure Ni NPs while the metallic state was the dominated phase in c-AuNi NCs. Additionally, the Au 4f XPS spectra of c-AuNi (Fig. 1d) suggests that the Au species are present in the metallic state and have a negative shift of 0.2 eV compared to that of pure Au NPs. The XPS results indicate that there are strong interactions between Au and Ni, and certain AuNi alloy formation at the interface of Au core and Ni shell.

**Fig. 2:**
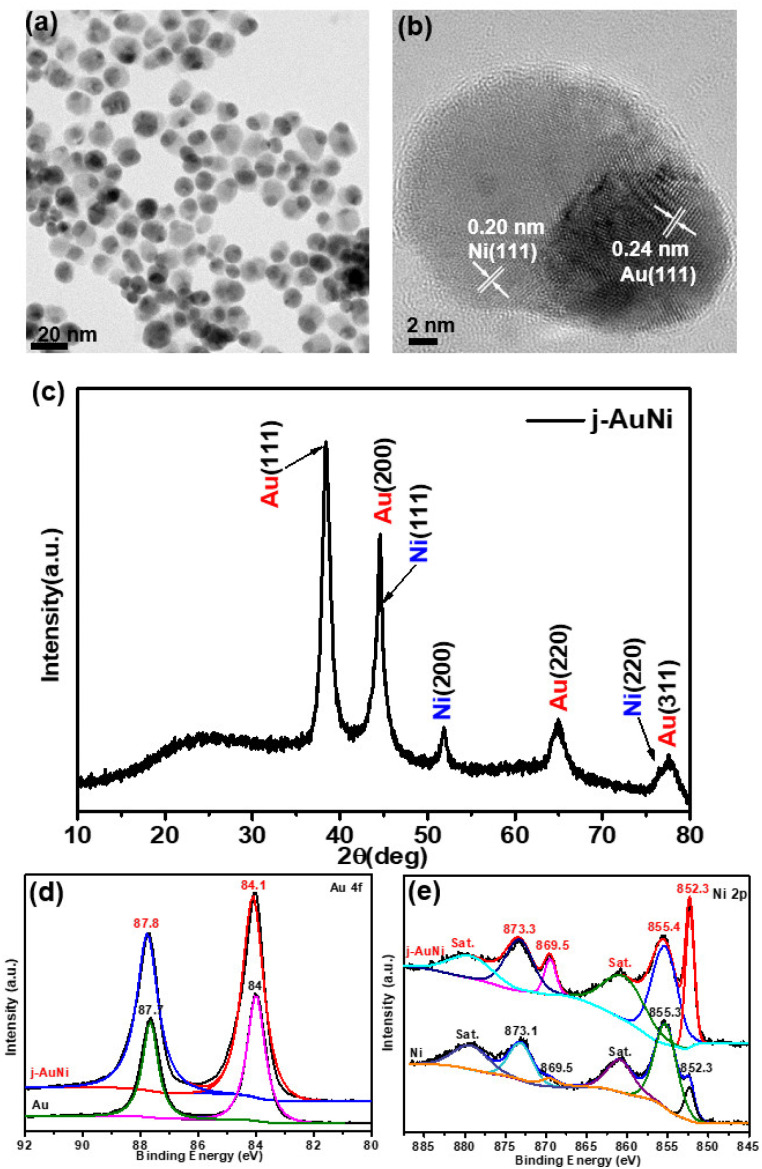
(a) TEM image, (b) HR-TEM image, and (c) XRD pattern of j-AuNi NCs, (d) Au 4f XPS spectra, and (e) Ni 2p XPS spectra of as-synthesized Au NCs Ni NPs and j-AuNi NCs.

For the j-AuNi sample made by using TOP as the passivation ligand, the TEM images (Fig. 2a and b) reveal a Janus structure that has one side as Au side and another side as Ni, as confirmed by the EDS mapping and line scan results (Fig. S2†). The molar ratio of Ni to Au was estimated as 2.3 : 1 based on the quantitative EDS analyses. The XRD pattern (Fig. 2c) appears the same as that of c-AuNi, showing bimetallic AuNi. In a typical high-resolution TEM (HRTEM) image (Fig. 2b), the lattice space of 0.24 nm for the tip and 0.20 nm for the tail correspond to Au (111) and Ni (111) planes, respectively.

Similar with the XPS Ni 2p spectrum of c-AuNi NCs, the two dominated peaks at binding energy 852.3 eV and 855.4 eV are correspond to metallic nickel and nickel hydroxide (Fig. 2e). However, the Au 4f peak of j-AuNi (Fig. 2d) shift to higher energy (0.3 eV) compared to that of c-AuNi. The possible reason is that the stronger bind of TPP with Au increases the electrons density of Au species in c-AuNi, which is further confirmed by the presence of XPS P 2p peaks at a 131.6 eV (Fig. S3†) in c-AuNi.^34^

### AuNi NCs formation mechanism

**Fig. 3:**
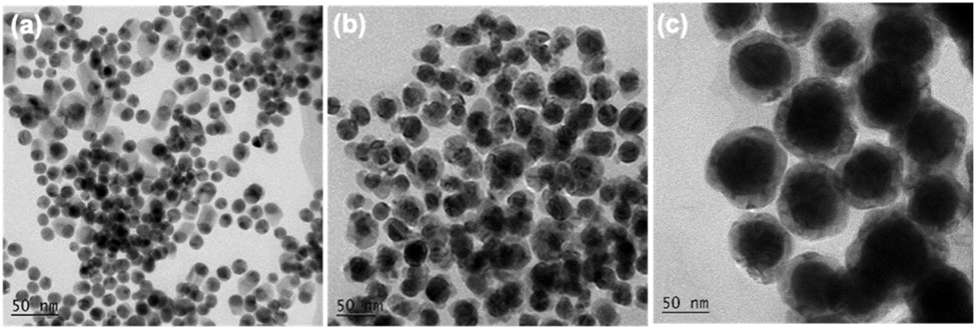
TEM images of the samples obtained with different amount of TPP: (a) 0.1 mmol, (b) 0.2 mmol, (c) 0.6 mmol.

In the synthesis of bimetallic AuNi NCs, it was found that the amount and type of ligands are crucial parameters affecting the geometry and size of final samples. If no TPP was used in the synthesis, almost all of the obtained particles were Au NCs with the size less than 10 nm (Fig. S4†). This result implies that Ni(acac)_2_ is hardly reduced to metallic Ni in OAm at 185 °C even in the presence of Au NPs seeds, which is consistent with previous reports.^35^ When 0.1 mmol TPP was introduced into the reaction, most of as-synthesized particles were spherical Au NPs with an average diameter of ∼15 nm and a few of them was coated with irregularly shaped Ni, as shown in Fig. 3a. This suggests that the presence of TPP can not only affect the size of Au but also assist the reduction of Ni^2+^.

As such, when the amount of TPP was increased to 0.2 mmol, most of obtained particles (Fig. 3b) showed core–shell structures with an average Au core size of ∼28 nm despite the Ni shell was not uniform. The critical value of 0.2 mmol TPP indicated that TPP not only served as a surface protection agent, but also acted as a coordination ligand directly involved in the reduction reaction. According to previous studies, TPP can easily react with HAuCl_4_ through reaction (1) and (2) to form stable complex Au(PPh_3_)Cl, which is white crystals and soluble in most organic solvents (TPP = PPh_3_).^36^ In addition, the larger size of obtained Au NPs (∼30 nm) and the appearance of white precipitates in the synthesis of Au NPs with the addition of TPP, confirm the formation of Au–TPP complex (see Fig. S5†). It also suggests that the successful formation of AuNi core–shell NCs would require molar ratio of TPP to HAuCl_4_ over 2 in order to inhibit fast nucleation of HAuCl_4_ in OAm and to achieve controllable Au seeds growth process dominated by thermal reduction of Au(PPh_3_)Cl.

1 AuCl_4_^−^ + PPh_3_ → Au(PPh_3_)Cl_3_ + Cl^−^

2 Au(PPh_3_)Cl_3_ + PPh_3_ → Au(PPh_3_)Cl + PPh_3_Cl_2_

With further increasing the amount of TPP, the obtained core–shell NCs exhibited larger Au core size and more uniform Ni shell (Fig. 3c) because higher concentration of TPP would decrease the reduction potential of Au(PPh_3_)Cl complex, thus further slowing down the nucleation and growth process of Au NPs.^35^ Moreover, the extra benzene-containing TPP ligands could enhance the electron cloud density on the surface of Au seeds, and therefore help to form uniform Ni shell.

In addition to the key factor of TPP, we have also investigated the effect of the Ni precursor amount on the size and morphology of obtained core–shell NCs. As shown in Fig. S6,† when a higher amount of Ni(acac)_2_ (0.6 mmol) was applied with other conditions unchanged, the obtained AuNi core–shell NCs exhibited more uneven and thicker Ni shell. In contrast, by decreasing the Ni(acac)_2_ amount to 0.2 mmol, most particles remain core–shell structure with a thinner shell of Ni (∼6 nm). This result implied that the thickness of Ni shell could be fine-tuned by varying the ratio of HAuCl_4_ to Ni(acac)_2_. Nevertheless, too large or too small amount of Ni precursor could lead to nonuniform shell.

**Fig. 4:**
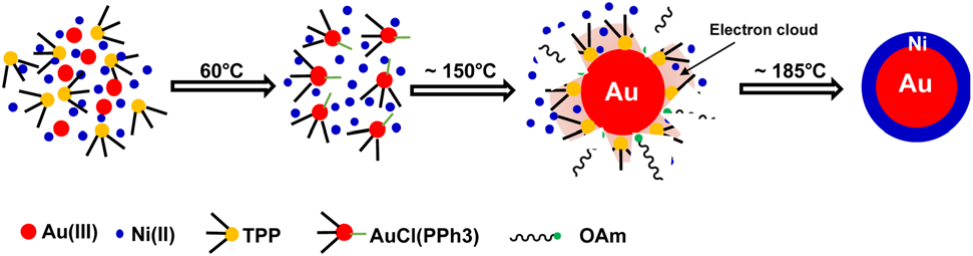
Schematic illustration of the synthetic process of c-AuNi NCs.

Based on previous studies and above experimental results, the formation mechanism of c-AuNi NCs could be illustrated in Fig. 4. After the mixing HAuCl_4_, TPP and Ni(acac)_2_ in OAm, the Au(PPh_3_)Cl complex would be produced as a new precursor of gold. Once Au(PPh_3_)Cl was reduced to Au NCs, its surface will be tightly bonded with a protective layer of TPP ligands, which could offer electrons rich environment for Au seeds due to the benzene groups of TPP. The TPP-capped Au NCs with high density of electrons greatly promoted the heterogeneous nucleation and subsequent growth of Ni on its surface. Furthermore, the capping effect of TPP could also reduce the high interfacial energy caused by the large lattice mismatch between Au and Ni, which favours the growth of Ni shell.^30^

**Fig. 5:**
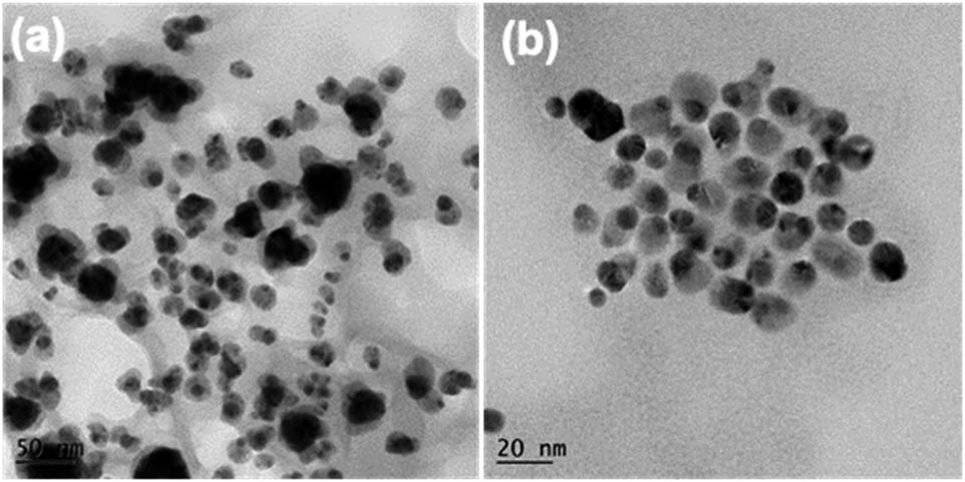
TEM images of the samples synthesized with different amount and type of ligands: (a) 0.4 mmol TPP, (b) 0.2 mmol TOP.

Compared with c-AuNi NCs, the formation process of j-AuNi NCs was more straightforward. In a typical synthesis, Au NCs were initially formed through the reduction of HAuCl_4_ in OAm. After introducing TOP, the sequential nucleation and growth of Ni commenced on the surface of gold NCs at elevated temperature upon thermal reduction of Ni–TOP complex by OAm. It has been reported that the TOP can coordinate with Ni^2+^ easily and Ni–TOP complex can be reduced in OAm at lower temperature than that of Ni(acac)_2_.^37,38^ Whereas, if 0.4 mmol TPP instead of 0.4 mmol TOP was used, we obtained irregular Au NCs with random coating of Ni shell (Fig. 5a) because the stronger capping ligands TPP could replace OAm absorbed on the performed Au NP surface but could not densely pack on its surface due to the large steric effect of phenyl group, which resulting in the aggregation of performed Au NCs. On the other hand, the large density electron cloud provided by TPP would induce the nucleation of Ni on multiple active surface sites of Au NCs, which leads to random coating of Ni shell.

**Fig. 6:**
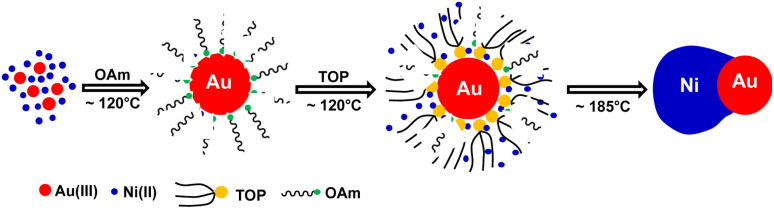
Schematic illustration of the synthetic process of j-AuNi NCs.

In comparison, when using TOP, it can more uniformly bind on preformed Au surfaces. Whenever there was one active surface point serving for Ni–TOP complex adsorption and reduction. The nucleation of created Ni^0^ atoms would deplete the Au surface electrons so that the remaining electron-deficient facets of Au are less active towards the nucleation of Ni. Subsequent quick growth of Ni occurring at this unique nucleation site would lead to formation of Janus AuNi NCs. The formation process is illustrated in Fig. 6. When the amount of TOP was reduced to 0.2 mmol, in addition to j-AuNi NCs, some bare Au NCs without Ni adhering were observed, which indicated that 0.2 mmol TOP was not able to supply sufficient Ni–TOP complex to deposit Ni on all Au NCs. In addition, as shown in Fig. S7,† Janus AuNi NCs with different compositions were obtained by simply adjusting the amount of Ni(acac)_2_. The prepared c-AuNi and j-AuNi nanocrystals did not show any large specific crystal facets, which also indicated that the ligands TPP and TOP did not have preferential strong binding on any specific crystal facet under the presented synthetic conditions.

### Photocatalytic hydrogen production activity

In order to explore the catalytic activities of the obtained AuNi NCs for hydrogen evolution, we incorporated the as-synthesized AuNi NCs onto GCN to form AuNi/GCN nanohybrids for photocatalytic test in 10 vol% TEOA aqueous solution under visible light irradiation. TEM image of AuNi/GCN (Fig. S8†) shows that the AuNi NCs were successfully loaded and well distributed on the GCN surface.

**Fig. 7:**
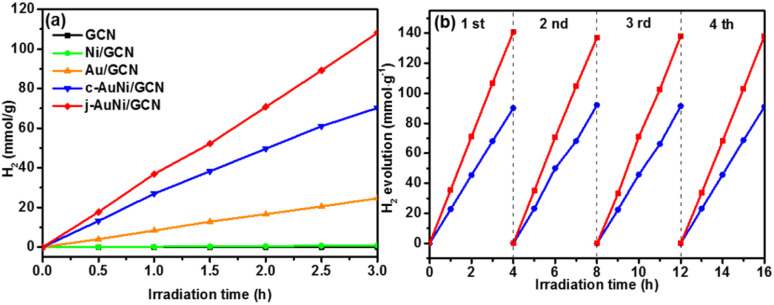
(a) Photocatalytic hydrogen production amount *versus* time over different metal NCs loaded GCN samples under visible-light irradiation; (b) cycling test results of c-AuNi/GCN and j-AuNi/GCN for H_2_ evolution. The H_2_ production amount is normalized by Au mass (g^−1^ Au).

Fig. 7a presents the photocatalytic hydrogen production amount *versus* time over different samples. By considering the cost of noble metal catalysts, the H_2_ production amount was normalized by Au mass (g^−1^ Au). The j-AuNi/GCN sample exhibits the best hydrogen evolution rate as 36 mmol g^−1^ h^−1^, which is much higher than that of Au/GCN (8.2 mmol g^−1^ h^−1^) and c-AuNi/GCN (23.4 mmol g^−1^ h^−1^). The enhanced HER activities for AuNi NCs as compared to pure Au could be attributed to the interactions between Au and Ni. Especially at the metallic Au–Ni interface, dealloying Ni from an AuNi alloy structure could lead to formation lower coordination Au sites, which shifts the d-band center toward the Fermi energy with stronger hydrogen binding capability towards higher HER activities.^39,40^

The sample stabilities were verified through cycling test. As shown in Fig. 7b, the hydrogen evolution activities over c-AuNi/GCN and j-AuNi/GCN were well preserved after four cycle test. The XRD patterns (Fig. S9 and S10†) showed no change for the samples after the cycling test. As for the XPS results (Fig. S11†), the peak of Ni 2p3/2 at 852.3 eV dropped due to transformation of some metallic Ni into NiO on the AuNi NC surface after long-term reaction. Accordingly, the Au 4f peak shift slightly to lower energy (0.2–0.3 eV) as compared to that of fresh AuNi NCs. Despite the slight structure change, we could still observe maintained H_2_ evolution activity during cycling test probably because the creation of NiO–Ni interface could also result in enhanced HER activity.^39^

**Fig. 8:**
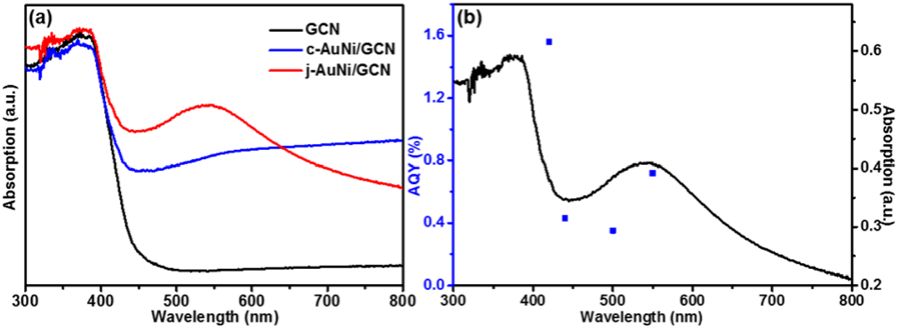
(a) UV-vis absorption spectra of the samples; (b) the AQY at different light wavelengths over j-AuNi/GCN.

As shown in Fig. 8a, compared to pure GCN and c-AuNi/GCN, j-AuNi/GCN exhibits a broad absorption peak at around 540 nm due to the localized surface plasmon resonance (LSPR) of the Au part. It is known that when plasmonic metal NPs are combined with excited semiconductor, synergistic energy transfer and charge migration between them would dramatically promote the photocatalytic processes.^41^ The LSPR excitation of the Au part of j-AuNi can significantly enhance the visible light absorption of GCN nearby j-AuNi NCs, which could greatly boost the photocatalytic activity of j-AuNi/GCN. To verify the LSPR effect, we measure the AQY of j-AuNi/GCN under different wavelengths. As indicated in Fig. 8b, the variation tendency of AQY is well consistent with the UV-vis absorption spectrum. The AQY for j-AuNi/GCN was estimated as 1.56% at 420 nm and still can reach 0.72% at 550 nm, which are comparable to the values of similar GCN-based photocatalysts in literature (Table S1†).

**Fig. 9:**
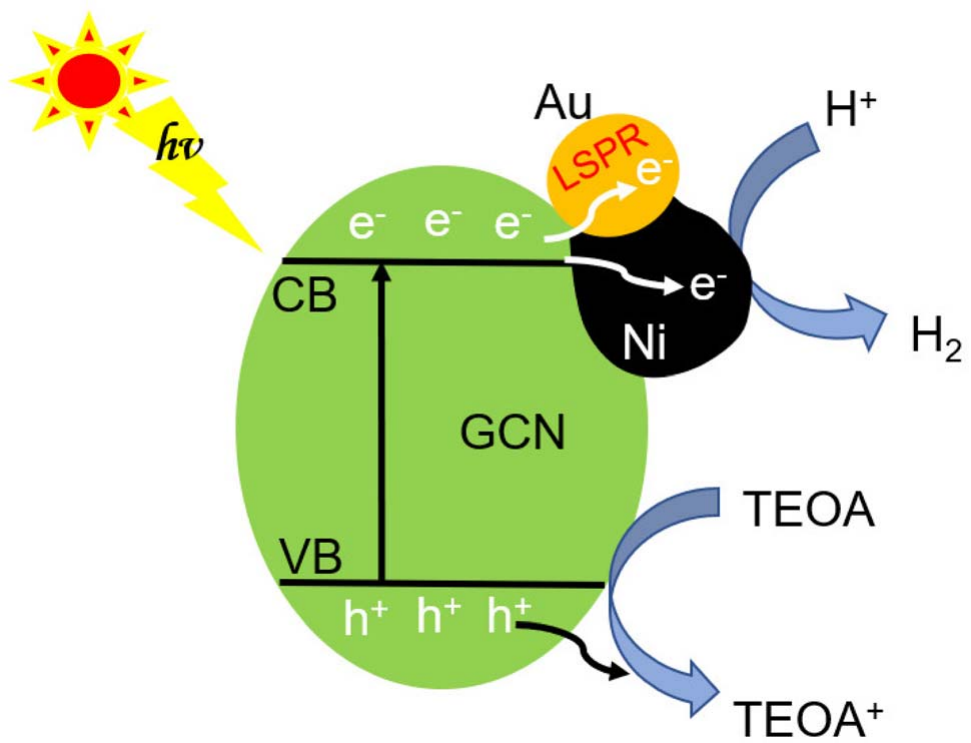
Schematic illustration of the photocatalytic processes of j-AuNi/GCN in TEOA solution.

The inherent GCN band structure did not show noticeable change upon loading of AuNi NCs. Based on the UV-Vis spectrum of GCN (Fig. 8a) and its electrochemical Mott–Schottky plots (Fig. S12†), the conduction band (CB) and valence band (VB) levels could be estimated as −1.12 V and 1.71 V (*vs.* NHE), respectively. When forming Schottky junction between GCN and AuNi NCs, the metallic Ni part of AuNi could serve as additional electron sink and receive the photogenerated electrons from excited GCN to promote charge separation, and provide more active surface sites for H^+^ adsorption and reduction into H_2_, as illustrated in Fig. 9.

## Conclusions

In conclusion, we developed a facile one-pot approach to synthesize AuNi Janus and core–shell NCs with uniform shape, tunable size and compositions. Systematic investigation on the structural evolution of bimetallic NCs revealed that the adding sequence and relative amount of different phosphine ligands (TPP or TOP) are crucial for the controllable formation of c-AuNi and j-AuNi. Moreover, the bimetallic AuNi/GCN nanohybrids exhibited much higher photocatalytic activity for hydrogen generation than Au/GCN and Ni/GCN due to the synergistic enhancement of two metals. In particular, the high activity of j-AuNi/GCN could be attributed to the enhanced light absorption caused by the LSPR effect of Au and the efficient charge separation arising from the electron-sink effect of Ni.

## Conflicts of interest

There are no conflicts to declare.

## Supplementary Material

RA-013-D2RA06325E-s001
