# Supplementary material for: Ligand-assisted morphology regulation of AuNi bimetallic nanocrystals for efficient hydrogen evolution
Source: RSC Adv. 2023 Jan 4;13(2):1229–35. doi: 10.1039/d2ra06325e (PMC9812016; doi:10.1039/d2ra06325e)
Supplement: RA-013-D2RA06325E-s001 [file RA-013-D2RA06325E-s001.pdf]

Electronic Supplementary Material (ESI) for RSC Advances.
This journal is © The Royal Society of Chemistry 2022

Supporting Information

Ligand-Assisted Morphology Regulation of AuNi Bimetallic Nanocrystals for Efficient Hydrogen Evolution

Chu Zhang, Can Xue

School of Materials Science and Engineering, Nanyang Technological University, 50 Nanyang avenue, 639798 Singapore.

Synthesis of Au NPs

In a typical synthesis, 0.1 mmol $HAuCl_4$ dispersed in 0.2 ml ethanol was injected to a round-bottom flask containing 7 ml OAm at 120 ^oC under vigorous stirring. Upon injection, the solution turned black red in colour over a period of one minutes, indicating the nucleation of Au. The solution was kept stirring at this temperature for 1 h. After cooling down to room temperature naturally, the mixture was precipitated by excess ethanol, which was isolated and collected via centrifugation. The obtained precipitate was then washed with a mixture of hexane and ethanol, and redispersed in hexane.

Synthesis of Ni NPs

Typically, 0.5 mmol of $Ni(acac)_2$ and 0.5 mmol TOP were added to 7 ml of OAm at 80 ^oC. After completely dissolution of all reagents, the mixture was heated to 210 ^oC and kept at that temperature for 1h with continuous mechanical stirring. After cooling down naturally, the mixed solution was precipitated by ethanol, which was isolated and collected through centrifugation. The obtained precipitate was then washed with a mixture of hexane and ethanol, and redispersed in hexane.

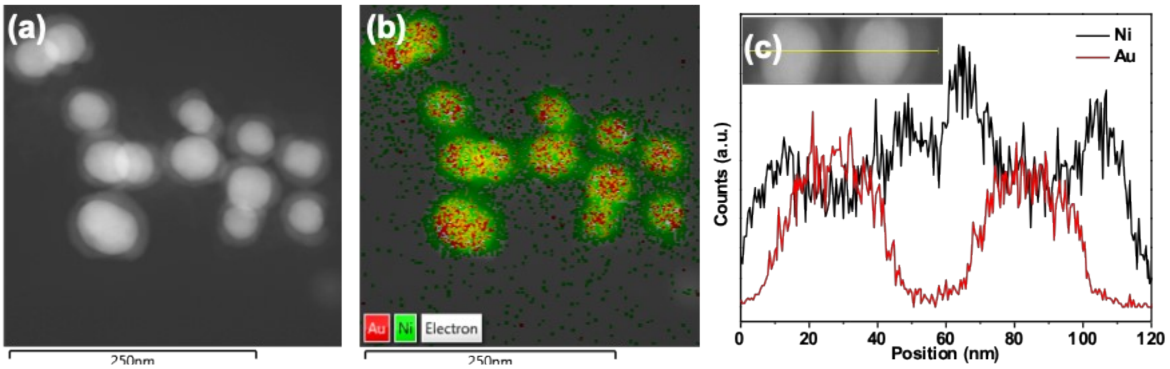

Fig. S1 (a) HAADF image, (b) EDS mapping, (c) EDS line scanning of c-AuNi NCs

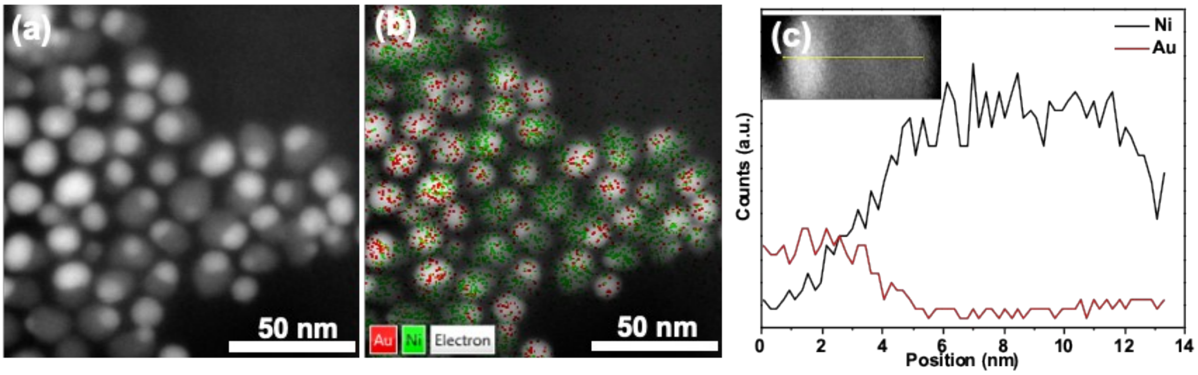

Fig. S2 (a) HAADF image, (b) EDS mapping, (c) EDS line scanning of j-AuNi NCs.

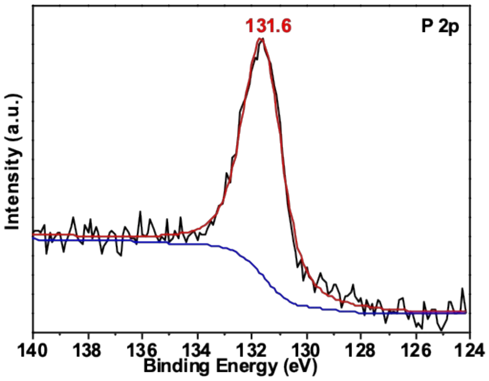

Fig. S3 P 2p XPS spectra of c-AuNi NCs.

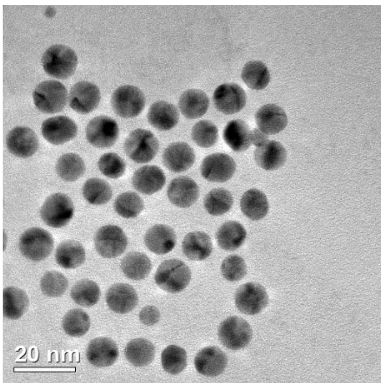

Fig. S4 TEM images of the AuNi NCs without TPP

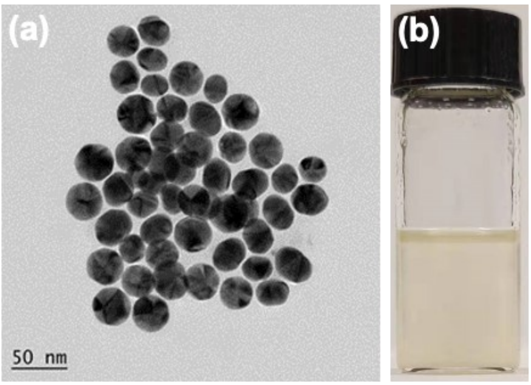

Fig. S5 (a) TEM images of Au NPs with 0.2 mmol TPP, (b) photograph of white precipitates appeared in OAm.

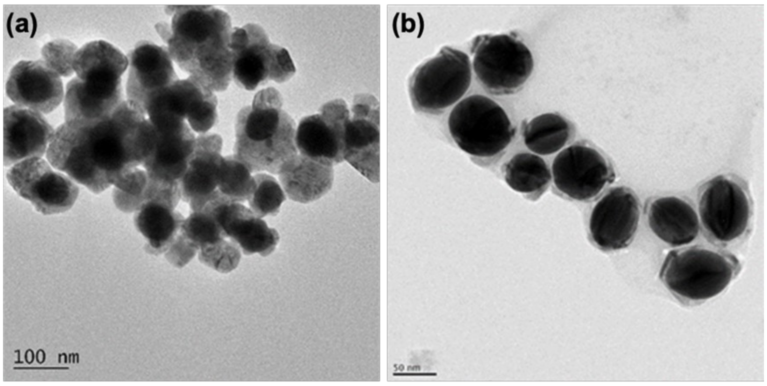

Fig. S6 TEM images of c-AuNi NCs formed with different amount of $Ni(acac)_2$. (a) 0.6 mmol, (b) 0.2 mmol

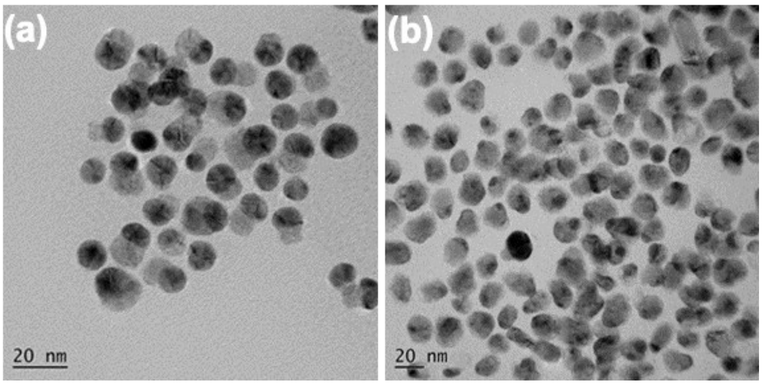

Fig. S7 TEM images of j-AuNi NCs formed with different amount of $Ni(acac)_2$. (a) 0.2 mmol, (b) 0.6 mmol.

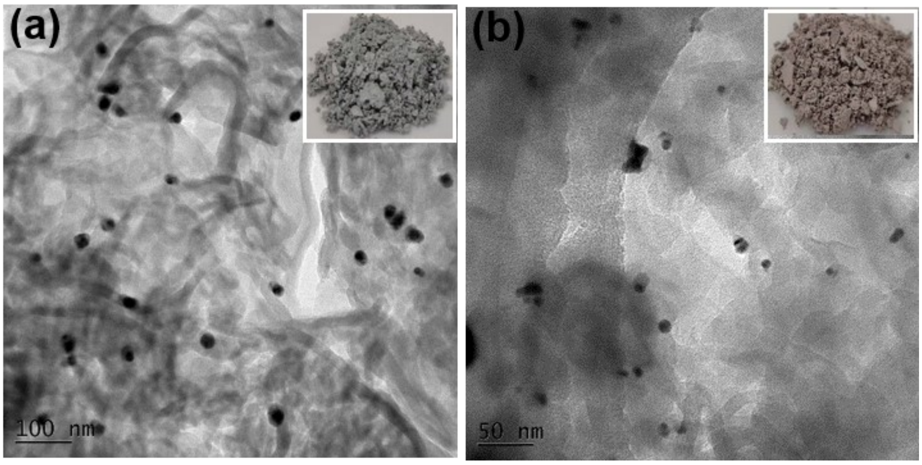

Fig. S8 TEM image of (a) c-AuNi/GCN, (b) j-AuNi/GCN

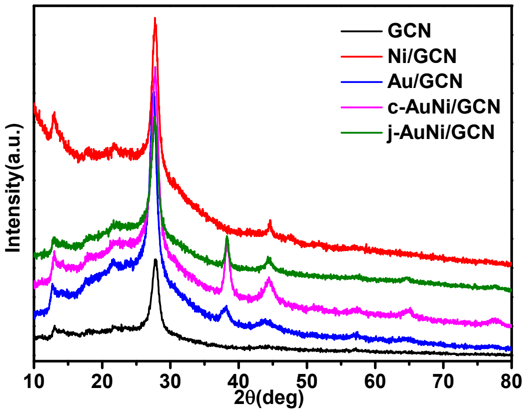

Fig. S9 XRD patterns of metal/GCN nanohybrids

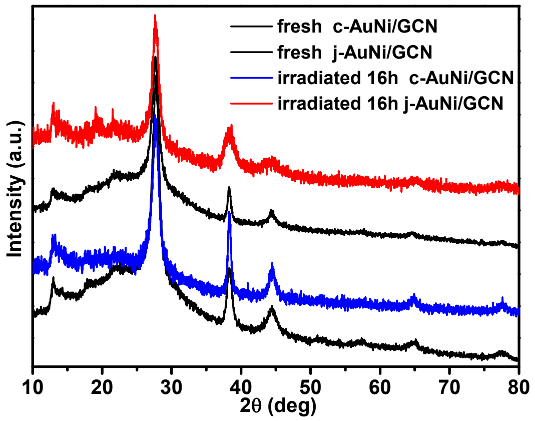

Fig. S10 XRD patterns of c-AuNi/GCN and j-AuNi/GCN before and after cycling test.

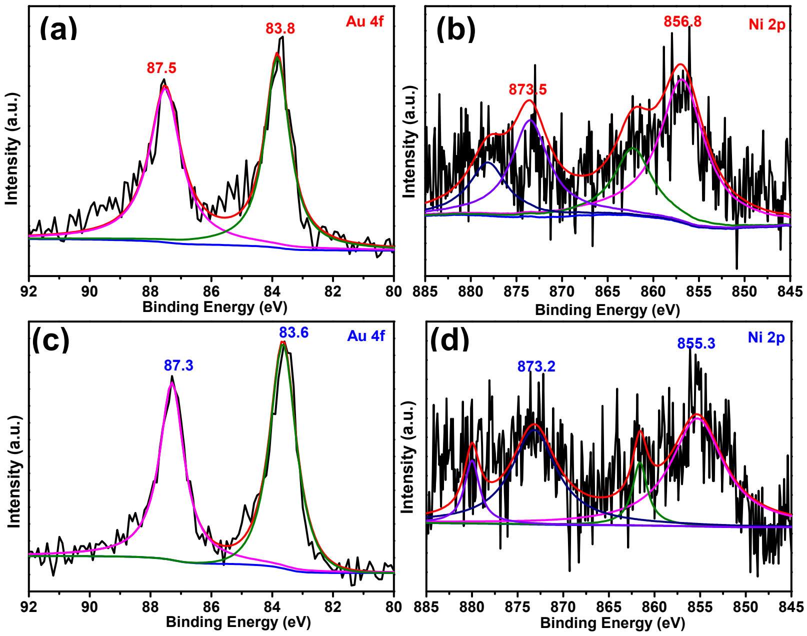

Fig. S11 (a) Au 4f XPS spectrum, (b) Ni 2p XPS spectrum of j-AuNi/GCN. (c) Au 4f XPS spectrum, (d) Ni 2p XPS spectrum of c-AuNi/GCN after cycling test

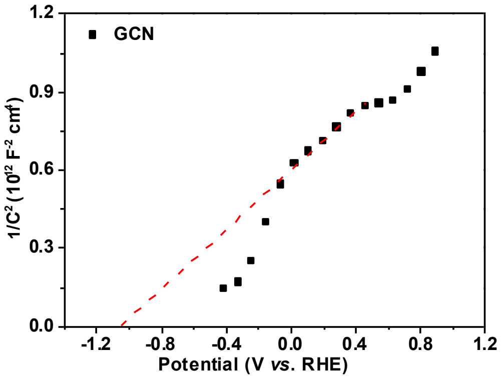

Fig. S12 The Mott-Schottky plot of GCN

Table S1. Comparison of AQY for photocatalytic H_2 evolution (in 10% TEOA) on GCN-based photocatalysts with different cocatalysts in the literatures

Cocatalysts	AQY	Ref.
Pt	3.01% at 450 nm<br>0.82% at 500 nm	ACS Appl. Mater. Interfaces 2020, 12, 47, 5260
NiS-Ag	1.21% at 420 nm	Chin. J. Catalysis, 2019, 40, 434
Ni	2.01% at 400 nm	J. Mater. Chem. A, 2016, 4, 9998
Ni_3N	2.3% at 420 nm	ACS Sustainable Chem. Eng. 2020, 8, 884
W_2C	1.52% at 420 nm	ChemSusChem, 2019, 12, 3355
AuNi	1.56% at 420 nm<br>0.72% at 550 nm	This work
